# Characteristics of Placental Histopathology in Women with Uncomplicated Pregnancies Affected by SARS-CoV-2 Infection at the Time of Delivery: A Single-Center Experience

**DOI:** 10.3390/biomedicines10123003

**Published:** 2022-11-22

**Authors:** Laura Sarno, Mariavittoria Locci, Caterina Fulgione, Francesca Perillo, Angela Dell’Isola, Dalila Mantelli, Cristina Sibillo, Gabriele Saccone, Giuseppe Maria Maruotti, Daniela Terracciano, Giuseppe Bifulco, Maurizio Guida, Maria D’Armiento

**Affiliations:** 1 Department of Neurosciences, Reproductive Science and Dentistry, University Federico II, 80131 Naples, Italy; 2 Pathology Unit, Department of Public Health, School of Medicine, University Federico II, 80131 Naples, Italy; 3 Department of Advanced Biomedical Sciences, University Federico II, 80131 Naples, Italy; 4 Department of Public Health, University Federico II, 80131 Naples, Italy; 5 Department of Translational Medical Sciences, University Federico II, 80131 Naples, Italy

**Keywords:** SARS-CoV-2, vascular malperfusion, placental histology

## Abstract

The aim of this study was, firstly, to analyze the histopathological characteristics of placentas in women with uneventful pregnancies and affected by COVID-19 at the time of delivery; and secondly, to correlate histological findings to maternal and neonatal characteristics. In our single-center prospective observational study, 46 placentas from term uncomplicated singleton pregnancies of patients with a documented SARS-CoV-2 infection at the time of delivery underwent histological examination. Despite a normal feto-maternal outcome, most of the placentas (82.6%) presented signs of maternal vascular malperfusion, while features of fetal vascular malperfusion were found in 54% of cases. No correlation was detected between maternal and neonatal characteristics and the severity of blood circulation disease, and abnormal findings were also described in asymptomatic patients. Moreover, we did not find any maternal symptoms or clinical details allowing for the prediction of abnormal placental findings in pregnancy complicated by COVID-19 infection. Our results suggest that SARS-CoV-2 infection during pregnancy could lead to acute placental dysfunction.

## 1. Introduction

Since March 2020, the SARS-CoV-2 pandemic has posed an outstanding emergency for national health systems worldwide, and one of the main concerns during COVID-19 pandemic has been the impact of this infection on pregnancy outcome.

During the first wave of the pandemic, different meta-analyses reported controversial results regarding the association between COVID-19 and perinatal outcome [1,2]; subsequently, emerging evidence highlighted the increased incidence of pregnancy complications, such as preeclampsia, preterm birth, Cesarean section, and stillbirth, in pregnancy complicated by COVID-19 [1,2,3,4,5,6,7,8,9].

Khalil et al. demonstrated a four-fold increase of stillbirth rate in a cohort of Britain pregnant women affected by COVID-19 [10]. Moreover, a population-based study in the United Kingdom, including almost 350,000 women, reported a 2.1-fold increased risk of stillbirth in pregnancies complicated by COVID-19 [11].

Starting from this evidence, attention has been focused on the placenta in order to find specific viral patterns that could explain increased risk of adverse outcome. It has been stated that vertical transmission is quite uncommon, with a rate of transmission ranging from 0% to 9% [12], but we cannot exclude that SARS-COV-2 infection could lead to placental damage, even in the absence of vertical transmission.

The first paper reporting on placental histology in COVID-19 pregnancies was a Chinese article analyzing three placentas delivered from pregnant women with confirmed SARS-CoV-2 infection and concluding that there were no morphological changes in the placenta that could be related to the infection [13].

Afterward, different case series analyzing histological abnormalities in COVID-19 placenta were reported, but the data were quite controversial. Di Girolamo et al., in a meta-analysis including 56 studies and 1008 pregnancies, reported that, in pregnant women with SARS CoV-2 infection, a significant proportion of placentas showed histopathologic findings of placental hypoperfusion and inflammation [14]. On the contrary, according to more recent studies [15,16], COVID-19 did not seem to be associated with specific histopathological findings, and placental abnormalities were not increased in COVID-19 pregnancies compared to controls. However, these studies included a too heterogeneous population, either for timing of infection, the inclusion of maternal preexisting conditions, or pregnancy related complications. On the other hand, small series reported that, in the case of adverse outcome, a specific placentitis that is characterized by trophoblast degeneration, intervillositis, and massive perivillous fibrinoid deposits could be detected [17,18].

In this prospective observational study, we aimed to define the histopathological characteristics of placentas from women with a singleton uncomplicated pregnancy affected by SARS-CoV-2 infection at the time of delivery. Secondly, we aimed to correlate histological findings to maternal and neonatal characteristics.

## 2. Materials and Methods

### 2.1. Study Design and Study Population

This was a single-center prospective observational study conducted at the University Hospital of Naples Federico II. We included pregnant women affected by SARS-CoV-2 infection at the time of delivery admitted at the Department of Mothers and Child of University Hospital Federico II, Naples, Italy.

SARS-CoV-2 infection was confirmed by positive nasopharyngeal swabs analyzed by using Abbott Real-Time SARS-CoV-2 kit on the Alinity platform, as recommended by the manufacturer. Briefly, 800 µL of UTM was analyzed. Samples were considered to be positive with a Ct value on *n* and/or on *ORF1b* genes that was ≤37.

Only non-vaccinated patients with a positive nasopharyngeal swab at the time of delivery with an uneventful singleton pregnancy were included.

The exclusion criteria were twin pregnancies, pregnancy-related complications (hypertensive disorders of pregnancy, gestational diabetes, placental abruptio, preterm labor, fetal growth restriction, and abnormal Doppler velocimetry), and patients with a previous infection of SARS-CoV-2 but with a negative swab at the time of delivery.

### 2.2. Histological Examination

As per local protocol, all the placentae from mothers affected by COVID-19 at delivery underwent histological examination at the Unit of Pathological Anatomy of the Department of Onco-Hematology, Imaging and Morphological Diagnostics and Forensic Medicine, at the University Hospital of Naples Federico II.

All placentas, membranes, and umbilical cords were stored at 4 °C and then underwent fixation in 10% formalin. Before the sampling, photographs of maternal and fetal surface were taken, and then pathologists proceeded with gross evaluation and sampling of placenta, according to the Amsterdam Placental Workshop Group Consensus Statement [19].

In brief, a minimum of 4 blocks were submitted, including one block with a roll of extraplacental membranes from the placental margin to the rupture edge and two cross-sections of the umbilical cord; and at least three blocks with full-thickness section of placental parenchyma obtained from the central two-thirds of the disc and including one adjacent to the insertion site. Histologic technical procedures were performed as previously described [20]. Histologic evaluation was performed according to the Amsterdam Placental Workshop Group Consensus Statement by M.D. and reviewed by two pathologists to confirm the diagnosis, and placental weight was compared with the normal values adjusted for gestational age [21].

### 2.3. Data Collection

Histological, clinical, and anamnestic data were recorded on a dedicated dataset, while respecting the patients’ confidentiality. Data entering was double-checked by A.D. and M.R.D. Patients were divided according to the grade of blood circulation disease into three groups: absence of blood circulation disease, low/moderate blood circulation disease, and severe blood circulation disease. Low/moderate blood circulation disease was defined by the presence of at least one of the following accelerated villous maturation, distal villous hypoplasia, peripheral infarction or central infarction < 5%, hypertrophic decidual arteriopathy, and FVM (non-high grade, according to the Amsterdam Placental Workshop Group Consensus Statement). Severe blood circulation disease was defined by the presence of retroplacental hemorrhage, central infarction > 5%, severe fibrinoid arteriopathy with fibrinoid necrosis, and high-grade FVM [19].

### 2.4. Statistical Analysis

Data were reported as mean ± standard deviation for continuous variables and number (percentage) for categorical variables. Chi-squared and Fisher’s exact tests were used to compare categorical variables between groups, while an ANOVA test was used for continuous variables. The *p*-values < 0.05 were deemed to be statistically significant. All statistical analyses were carried out by using SPSS statistics 26.0 (IBM Corporation, Armonk, NY, USA).

## 3. Results

### 3.1. Study Population

Forty-six non-vaccinated patients who were affected by SARS-CoV-2 infection at the time of delivery, with a singleton uneventful pregnancy, were included in the study. Among them, only 1 patient (2.2%) required respiratory support upon admission; 12 (26.1%) presented mild symptoms, such as a sore throat, fever, and arthralgia; and the remaining 33 (71.7%) were asymptomatic.

None of the newborns had a positive nasopharyngeal swab postnatally. Moreover, no neonatal complications were reported, and none was admitted to the Neonatal Intensive Care Unit.

The characteristics of the included patients and their newborns are summarized in Table 1.

### 3.2. Histological Examination

The macroscopical characteristics of the placentas are summarized in Table 2.

In six cases, the placentas were small for gestational age (13%).

**Figure 1:**
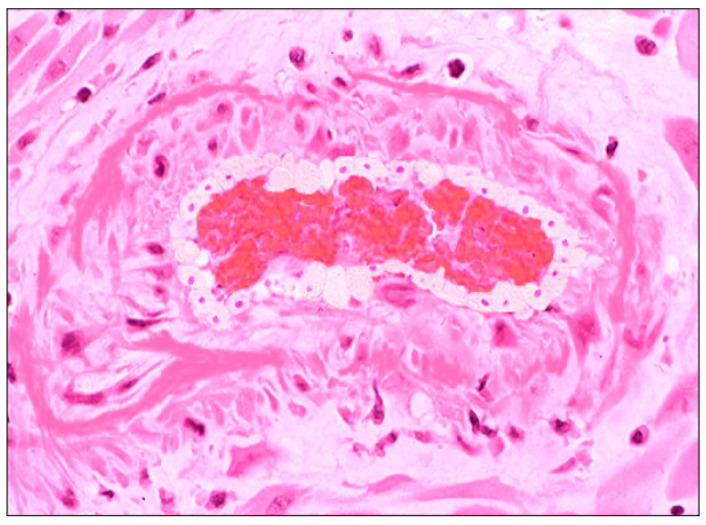
Signs of MVM in placenta from woman with SARS-CoV-2 infection: spiral artery atherosis with lipid laden macrophages (hematoxylin-and-eosin stain; original magnification 25×).

Features of maternal vascular malperfusion (MVM) were present in 38 cases (82.6%), including retroplacental hemorrhage (*n* = 7, (15.2%)), atherosis (Figure 1), fibrinoid necrosis of maternal vessels (*n* = 5 (10.8%)), peripheral infarctions (*n* = 12 (26%), and central infarctions (*n* = 5 (10.8%)).

**Figure 2:**
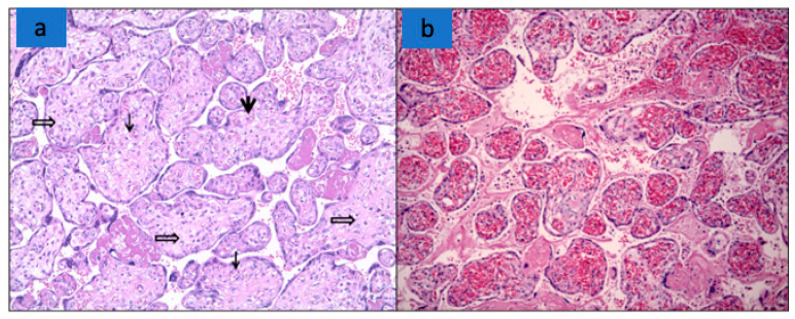
Signs of FVM in placenta from woman with SARS-CoV-2 infection. (**a**) Delayed villous maturation: increased number of large distal villi, with increased capillaries (**↓**), stromal macrophages (⇓), and diffused stromal oedema (hematoxylin-and-eosin stain; original magnification 10×). (**b**) Third trimester placenta with fibrosis of the villi and mural fibrin deposition (hematoxylin-and-eosin stain; original magnification 10×).

The features of fetal vascular malperfusion (FVM) were present in 25 cases (54%), including delayed villous maturation (*n* = 14 (30%)) (Figure 2), chorangiosis (*n* = 3 (6.5%)), villous edema (*n* = 13 (28%)), and mural fibrin deposition in fetal vessels (*n* = 1 (2%)) (Figure 2c).

One case (2.2%) showed acute/chronic mixed inflammatory infiltrate, as per low histologic chorioamnionitis. Twenty-two placentas (47.8%) showed areas of calcification.

**Figure 3:**
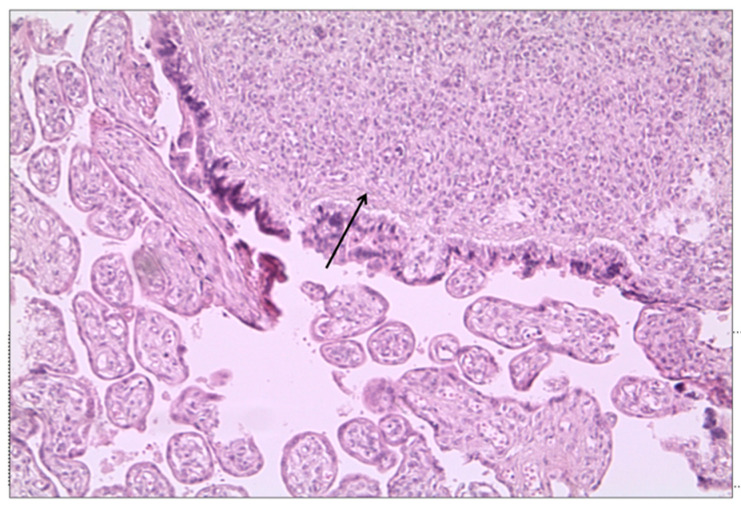
Placental chorioangioma in a woman with SARS-CoV-2 infection (→) (hematoxylin-and-eosin stain; original magnification 10×).

The placenta of the patient who required ventilatory support presented chorangiosis and subcentimetric chorioangioma (Figure 3).

Patients were divided into three groups according to the grade of blood circulation disease: absence of blood circulation disease (*n* = 4 (8.7%)), blood circulation disease of low/moderate grade (*n* = 25 (54.3%)), and blood circulation disease of severe grade (*n* = 17 (37%)). The maternal and neonatal characteristics stratified by groups are reported in Table 3. No statistically significant differences were reported among the groups.

## 4. Discussion

### 4.1. Summary of Study Findings

In our single-center prospective observational study, 46 placentas from term uncomplicated pregnancies of patients with a documented SARS-CoV-2 infection at time of delivery underwent histological examination. We included only patients without preexisting chronic conditions or pregnancy-related complications in order to exclude disorders that have been reported to be related to MVM and FVM. Despite a normal feto-maternal outcome, most of the placentas (82.6%) presented signs of MVM, including retroplacental hemorrhage, atherosis, and fibrinoid necrosis of maternal vessels, while features of FVM (delayed villous maturation, chorangiosis, villous edema, and mural fibrin deposition in fetal vessels) were found in more than half of cases.

No correlations were detected between maternal and neonatal characteristics and the severity of blood circulation disease, and abnormal findings were also described in asymptomatic patients, as previously reported [18,22]. Moreover, we did not find any maternal symptom or clinical detail allowing for the prediction of abnormal placental findings in pregnancy complicated by COVID-19 infection.

### 4.2. Interpretation of Study Findings and Comparison with the Literature

According to our study, placentas from patients with acute SARS-CoV-2 infection at delivery have a high rate of MVM and FVM features that have been previously reported to be associated with adverse feto-maternal outcome [23,24,25,26].

Even if these findings have also been reported in 10–50% of pregnancies with a healthy outcome [27], these features were more common in our series and could explain the increased risk of pregnancy-related complication (such as fetal death, preterm birth, preeclampsia, and emergency Cesarean delivery) reported in COVID-19 pregnancies [9,11].

None of the placentas in our study was tested for SARS-CoV-2 viral RNA or protein. Per hospital protocol, all live infants born to SARS-CoV-2 mothers were tested by a nasopharyngeal and throat swab at greater than 24 h of life for a minimum of once and up to twice for SARS-CoV-2, and none was positive, confirming that vertical transmission is uncommon, even though it has been reported to be possible [16,17]. However, a previous study reported variable degrees of histiocytic intervillositis, perivillous fibrin deposition, trophoblast necrosis, and fetal vascular malperfusion, even in absence of vertical transmission [28]. Therefore, perinatal mortality and morbidity could be not directly related to viral infection itself [22] but to the histological abnormalities observed in SARS-CoV-2 infection. Even if the etiology of placental vascular malperfusion in COVID-19 placentas should be further investigated, some authors concluded that the hypercoagulability associated with the infection can lead to these placental abnormalities [29]. According to other studies, placental hypoperfusion could be a secondary effect of the virus, leading to a compromission of maternal hemodynamic status [30] or to an aggressive inflammatory response [14].

However, as previously reported in the meta-analysis by Suhren et al. [16] that included 1452 placentas, we could not find a specific histopathologic pathway that could be suggestive of COVID-19 infection; consistently with previous studies [16], COVID placentas presented non-specific vascular malperfusion, even if existing data were quite different among series. Similar to our data, a small-for-gestation placenta was found in 15% of cases [16].

The presence of a non-specific pattern could be related to the exclusion of adverse perinatal outcome in our series [31]. An analysis of the placenta in cases of stillbirth and neonatal death concluded that SARS-CoV-2 could be related to extensive and specific placentitis, causing placental malperfusion and insufficiency that could lead to intrauterine and perinatal death [31,32].

### 4.3. Study Strength and Limitation

The strength of our study was the selection of physiologic patients with a full-term pregnancy and the exclusion of other pre-existing chronic conditions or pregnancy-related complications leading to placental abnormalities that have been included in previous studies [29,33,34,35,36,37,38,39,40,41,42], making the recognition of the effect of SARS-CoV-2 on the placenta difficult. Moreover, not all previous published studies about placental histopathology in COVID-19 pregnancies included maternal characteristics.

A limitation of our study was the absence of a control group. However, te rate of FVM and MVM seems to be higher than the one reported in uneventful pregnancy [27]. Moreover, the sample size is limited, and results are preliminary and should be confirmed by a larger study.

### 4.4. Clinical and Research Implications

The finding of histopathologic placental abnormalities in patients with a recent infection (a positive nasopharyngeal swab at time of delivery) enforces the idea that COVID-19 can lead to acute and rapid placental dysfunction [18,22].

These findings suggest that increased antenatal surveillance for women with COVID-19 may be warranted, especially for pregnancies at term, for whom an expectant management could be useless.

Even if we had a normal short-term outcome, we cannot exclude long-term sequelae in these offspring. Indeed, it has been reported that COVID-19 could lead to maternal inflammation and oxygen deficiency, with consequences on the neurodevelopment of the newborns [16,43].

## 5. Conclusions

Placentas from patients testing positive for SARS-CoV-2 at the time of delivery presented signs of MVM and FVF, enforcing the idea that COVID-19 could lead to acute placental dysfunction. Histologic abnormalities did not seem to be related to the severity of maternal pathology or to maternal characteristics. Further studies comparing placentas in COVID-19 pregnancies with normal versus adverse outcome and including long-term outcomes of the offspring should be warranted.

## Figures and Tables

**Table 1:** Characteristics of the included patients.

Variables	
Maternal age (years)	29.87 ± 4.9
Gestational age at admission (weeks)	39.102 ± 1.16
Gestational age at delivery (weeks)	39.316 ± 1.09
Interval between first positive swab and delivery (days)	3416 ± 3315
Parity	
Primiparity	12 (26)
Multiparity	34 (74)
Gravidity	2.25 ± 1.04
S0_2_ at admission	97.2 ± 2.5
Needing for respiratory support	1 (2.2)
Diastolic blood pressure at admission (mmHg)	70.6 ± 8.89
Systolic blood pressure at admission (mmHg)	122.17 ± 4.67
Type of delivery (*n* (%))	
Spontaneous	21 (46)
Cesarean section	25 (54)
Indication for CS (*n*%)	
Previous CS	13 (52)
Failure of induction	3 (12)
Labor dystocia	6 (24)
Maternal request	2 (8)
Breech presentation	1 (4)
Birthweight (grams)	3234.2 ± 405
APGAR at 1 min	7.75 ± 1.05
APGAR at 5 min	8.94 ± 0.39
Fetal sex (*n* (%))	
Female	22 (47.8)
Male	24 (52.2)
Head circumference (cm)	33.52 ± 1.27
Length (cm)	48.7 ± 2.7

**Table 2:** Macroscopical characteristics of the placentas in patients affected by SARS-CoV-2 infection.

Characteristics	
Placental weight (grams)	460.46 ± 91.69
Maximum thickness (cm)	2.86 ± 0.60
Minimum thickness (cm)	1.78 ± 0.60

**Table 3:** Maternal and neonatal characteristics stratified by grade of blood circulation disease.

	Low/Moderate Blood Circulation Disease	Severe Blood Circulation Disease	Absent Blood Circulation Disease	*p*-Value
	*n* = 25	*n* = 17	*n* = 4	
Maternal age	30.32 ± 6.04	29.58 ± 4.29	28.25 ± 1.92	0.853
Gestational age at admission	38.633 ± 2.577	39.05 ± 1.5	38.99 ± 1.74	0.349
Gestational age at delivery	39.107 ± 1.01	39.4 ± 1.24	39.36 ± 1.55	0.248
Interval between first positive swab and delivery (days)	1.48 ± 0.897	2.31 ± 1.79	4.5 ± 4.09	0.08
Parity	1.5 ± 0.9	1.7 ± 1.2	2.3 ± 1.2	0.123
Gravidity	2.08 ± 0.9	2.06 ± 1.02	3 ± 1	0.197
S0_2_ at admission	95.6 ± 3.44	97.33 ± 1.37	98.25 ± 0.43	0.298
Needing for respiratory support	1 (4%)	0 (0%)	0 (0%)	--
Diastolic blood pressure at admission (mmHg)	68.8 ± 7.81	71.25 ± 8.56	70 ± 7.07	0.748
Systolic blood pressure at admission (mmHg)	121.93 ± 4.01	123.07 ± 6.05	122.5 ± 4.33	0.963
Presence of mild symptoms	7 (28)	4 (23.5)	1 (25)	0.129
Type of delivery				
Spontaneous	10 (40%)	8 (47%)	4 (100%)	0.06
Cesarean section	15 (60%)	9 (53%)	-	
Birthweight (grams)	3265.23 ± 393.84	3172.3 ± 445.64	3242.5 ± 299.02	0.903
APGAR at 5 min	8.9 ± 0.436	9 ± 0.39	9 ± 0.11	0.953
Head circumference (cm)	34.09 ± 0.94	33 ± 1.31	33 ± 1.0	0.296
Length (cm)	49.33 ± 1.43	48.11 ± 3.6	49.21 ± 2.45	0.727

## Data Availability

All the results obtained from the study were reported in the manuscript. There are no additional data.
